# Gait-ViT: Gait Recognition with Vision Transformer

**DOI:** 10.3390/s22197362

**Published:** 2022-09-28

**Authors:** Jashila Nair Mogan, Chin Poo Lee, Kian Ming Lim, Kalaiarasi Sonai Muthu

**Affiliations:** Faculty of Information Science and Technology, Multimedia University, Melaka 75450, Malaysia

**Keywords:** gait, gait recognition, deep learning, transformers, vision transformer, vit, attention

## Abstract

Identifying an individual based on their physical/behavioral characteristics is known as biometric recognition. Gait is one of the most reliable biometrics due to its advantages, such as being perceivable at a long distance and difficult to replicate. The existing works mostly leverage Convolutional Neural Networks for gait recognition. The Convolutional Neural Networks perform well in image recognition tasks; however, they lack the attention mechanism to emphasize more on the significant regions of the image. The attention mechanism encodes information in the image patches, which facilitates the model to learn the substantial features in the specific regions. In light of this, this work employs the Vision Transformer (ViT) with an attention mechanism for gait recognition, referred to as Gait-ViT. In the proposed Gait-ViT, the gait energy image is first obtained by averaging the series of images over the gait cycle. The images are then split into patches and transformed into sequences by flattening and patch embedding. Position embedding, along with patch embedding, are applied on the sequence of patches to restore the positional information of the patches. Subsequently, the sequence of vectors is fed to the Transformer encoder to produce the final gait representation. As for the classification, the first element of the sequence is sent to the multi-layer perceptron to predict the class label. The proposed method obtained 99.93% on CASIA-B, 100% on OU-ISIR D and 99.51% on OU-LP, which exhibit the ability of the Vision Transformer model to outperform the state-of-the-art methods.

## 1. Introduction

Gait is an emerging biometric that utilizes walking patterns to recognize an individual. Gait is a behavioral pattern which is difficult to imitate and impossible to conceal [1]. Other than that, the subjects’ cooperation is not needed to perform the identification using gait [2]. Due to these advantages, gait recognition is mostly used at banks, airports, and crime scenes. Nonetheless, the performance of the gait recognition is affected by various factors such as viewing angle, carrying condition, clothing, and walking speed.

During the initial stage, the gait recognition problem was tackled by using handcrafted techniques. The handcrafted methods can be categorized as model-based methods and appearance-based methods. Model-based methods [2,3,4,5,6] identify a gait by analyzing the motion information acquired from a human model, while the appearance-based methods [7,8,9,10,11,12,13,14] capture the gait parameters directly from the silhouettes. The model-based methods entail high computational costs as the model-based methods involve a human model to extract the features. On the other hand, appearance-based methods demand low computational costs and are easy to implement. Nevertheless, the handcrafted methods use manually extracted features to perform the recognition, where the significant features are omitted.

Deep learning methods [15,16,17,18,19,20,21,22] have garnered a great deal of interest among researchers. Deep learning algorithms can deliver high performance without the need for feature engineering, which is time-saving. In recent times, the application of self-attention mechanisms in deep neural networks has become popular due to the success of self-attention mechanisms in natural language processing applications such as speech recognition, language modeling, and machine translation. Self-attention is a mechanism where a single sequence is arranged in various positions to determine the representation of the sequence. Self-attention-based architecture, particularly, Transformers are well-known due to its adaptivity. However, the application of Transformers in computer vision, especially in gait recognition, is limited where the existing works are mainly adopting Convolutional Neural Network (CNN) architectures.

In view of this, this paper presents a gait recognition method with Vision Transformer (ViT), referred to as Gait-ViT. To begin with, gait energy image (GEI) is attained by averaging the gait silhouettes over a gait cycle. The obtained images are then divided into a fixed number of patches. Subsequently, the patches are converted to a sequence where patch embedding and positional embedding are applied. The embedded sequence of vectors is then sent to the Transformer encoder to encode the final representation. The first token of the sequence patches is then fed to the multi-layer perceptron for class prediction.

The main contributions of this work are specified below:

- The gait images are represented as GEIs to capture the significant limb movements in gait while suppressing the effects of noise, shadow, and incomplete silhouettes.
- The Vision Transformer model encodes prominent gait features on the strength of multi-head attention mechanism, receptive fields, layer normalization, global operation, and residual connections, which elevates the performance of the proposed method.
- The performance of the proposed Gait-ViT method is evaluated on three datasets, namely CASIA B, OU-ISIR D, and OU-LP datasets.

## 2. Related Works

The existing works in gait recognition can be broadly categorized into handcrafted methods, deep learning methods, and attention methods. Handcrafted methods manually design the features to represent the gait movements; subsequently, the features are passed into machine learning classifiers. The deep learning methods learn the complex features by hidden layers that serve different purposes, such as convolutional layers, pooling layers, fully connected layers, etc. In recent years, attention models have achieved outstanding performance in Natural Language Processing due to their ability to encode contextual significance in the input. In view of this, researchers set out to explore the adoption of attention models in computer vision applications.

### 2.1. Handcrafted Approach

Handcrafted methods can generally be divided into two groups, namely model-based and appearance-based methods. The model-based methods build the human skeleton model to extract the motion features. In earlier days, Wang et al. (2016) [23] built a human skeleton model, which captured the 3D coordinates of 21 joints. Static features and dynamic features were then extracted using the 3D coordinates. The experiments on their self-collected dataset with 52 subjects recorded a correct classification rate of 92.3%. Zhen et al. (2018) [24] utilized joint angles, joint elevation, and stride widths as dynamical information and structural information. Radial Basis Function was employed to capture the variations of gait sequences over deterministic learning. The proposed method achieved an accuracy of 77.6–90.7% with different features on the multi-view Kinect-based Gait database. Choi et al. (2019) [25] presented a frame-level matching, which reduces the effect of noisy silhouettes. Other than that, a weighted majority voting was introduced, which allocates different weights for every frame. The classification was performed by applying weighted majority voting through frame-level scores. On their self-collected gait datasets, the method obtained an average recognition rate of 89.26%. Lima et al. (2021) [26] used PoseDist and PoseFrame for gait recognition problems. The coordinates of each subject were identified using pose estimation. The obtained coordinates were then fed to PoseDist and PoseFrame. The PoseDist captured the features from pose joints and then classified them using nearest neighbors, while the PoseFrame extracted and normalized the coordinates of joints and fed them into a multi-layer perceptron for classification. The highest average rank-1 score of 97.97% was yielded by the PoseFrame on the CASIA dataset A.

Unlike the model-based methods, appearance-based methods utilize the features obtained from silhouettes after background subtraction. Rida et al. (2016) [27] used the Statistical Dependency feature selection algorithm and Globality-Locality Preserving Projections algorithm to reduce the effect of different walking conditions. The 1-nearest neighbor was employed to classify the subjects. The method achieved an average correct classification rate of 86.06% on the CASIA dataset B. Mogan et al. (2017) [28] encoded the direction of gait sequence and temporal patterns by integrating motion history image, binarized statistical image features, and histograms of oriented gradients. The class label was determined by the minimum Euclidean distance between the gallery and probe sequences. A recognition rate of 93.42% was obtained on the CASIA dataset B. Wang et al. (2018) [29] extracted various orientation and scale information from the Gabor wavelet to produce a gait energy image. The feature dimension reduction was performed using a two-dimensional principal component analysis technique where the inter-class distance was increased, and the intra-class distance was reduced. The subjects were classified using Support Vector Machine (SVM). The method recorded an average correct classification rate of 93.52% on the CASIA dataset B. Arshad et al. (2019) [30] employed Quartile Deviation of Normal Distribution to capture the motion patterns from the gait sequence. Shape and texture features were extracted from the motion information. Both the features were then fused using the Bayesian model, and the top features were chosen using Binomial Distributions. The method yielded an 87.7% accuracy on the CASIA dataset B.

### 2.2. Deep Learning

Deep learning, also referred to as deep neural networks, consists of numerous hidden layers that exhibit the network’s depth. The deep learning methods learn salient features directly from the input without the need to identify the features manually. CNN is one of the popular architectures among deep learning methods where the features and patterns within an image are detected.

Wolf et al. (2016) [31] developed a 3D convolutional neural network with seven convolution layers, six max-pooling layers, two fully connected layers, and a classification layer along with a unique input format, which consists of grayscale and optical flow to reduce the effects of color invariance. On the CASIA dataset B, the model obtained accuracy in the range of 94.3–99.9% at different viewing angles. Subsequently, Wang et al. (2020) [32] proposed a new gait representation called trituple gait silhouettes (TTGS) by combining three consecutive gait silhouettes. Along with that, a multichannel convolutional network was developed, which accepts the TTGS members simultaneously. The method achieved a rank-1 score of about 65% on the CASIA dataset B and 68% on the OU-LP dataset. Subsequently, Su et al. (2020) [33] presented center-ranked loss to incorporate the positive and negative samples. The center-ranked loss was assessed using a network, which comprises seven convolution layers, three max-pooling layers, and a fully connected layer. The method recorded an average recognition accuracy of 74.8% and 57.8% on the CASIA dataset B and OU-MVLP dataset, respectively. Song et al. (2019) [34] built a network that contains two CNNs, where the first CNN segments the gait silhouettes and the other CNN performs gait classification. Other than that, joint learning techniques were applied to ensure the network could handle noisy silhouettes and complex conditions. On the CASIA dataset B, the method yielded a rank-1 mean accuracy of 89.9%.

Ding et al. (2021) [35] explored the standard CNN to encode the motion information. A behavioral information extractor (BIE) was employed to determine the association among the frames in the time axis based on motion templates, while a multi-frame aggregator (MFA) was utilized to integrate and condense the features for classification. The experiments on the CASIA dataset B (normal subset) and OU-MVLP dataset demonstrated the method achieved 95.2% and 83.8% mean accuracy, respectively. Recently, Mogan et al. (2022) [36] incorporated a pre-trained DenseNet-201 model and multilayer perceptron for gait recognition problems. The pre-trained DenseNet-201 model was utilized to extract the gait features, while the multilayer perceptron was used to encode the association among the obtained features and the class labels. The model obtained a recognition rate of 99.17% on the OU-LP dataset. Mogan et al. (2022) [37] fine-tuned a pre-trained VGG-16 model to learn low-level and intricate gait features. The relationship between the acquired features and the subjects was determined using a multilayer perceptron. On the OU-LP dataset, the model recorded an accuracy of 99.10%.

### 2.3. Attention Models

In recent times, the incorporation of transformers in convolutional networks has been applied for gait recognition. Transformers utilize an attention mechanism that targets specific parts of an input to improve performance. Li et al. (2019) [38] presented a CNN-based joint intensity transformer network to reduce the effect of clothing and carrying conditions on gait recognition. The network was made up of a joint intensity metric estimation net, a joint intensity transformer, and a discrimination network. The model obtained a rank-1 score of 74.03% on the OU-LP-Bag dataset. Similarly, Xu et al. (2020) [39] proposed CNN-based architecture, which comprised a pairwise spatial transformer (PST) followed by a recognition network. A pair of inputs from various views were fed into the network. The PST estimated and transformed the features of the input pair into an intermediate view between the input pair views. The recognition network was used to determine the dissimilarity score of the matching pair. The proposed model achieved a mean rank-1 identification rate of 59.9% on the OU-MVLP dataset. Wang and Yan (2021) [40] incorporated non-local features and regionalized features to capture the intrinsic gait features. A network with two channels that accepts two inputs were developed. The classification was performed using a self-attention mechanism. The network recorded a mean recognition rate of 91.7% on the CASIA dataset B with 36°.

Most of the existing works are based on CNNs due to their high performance. However, CNNs have less access to global information at the lower layer, which results in the loss of information. To this end, this work leverages the ViT model due to its ability to propagate the information clearly from a lower level to a higher level. Moreover, ViT model accumulates the global information early with the help of the self-attention mechanism.

## 3. Gait Recognition with Vision Transformer

**Figure 1:**
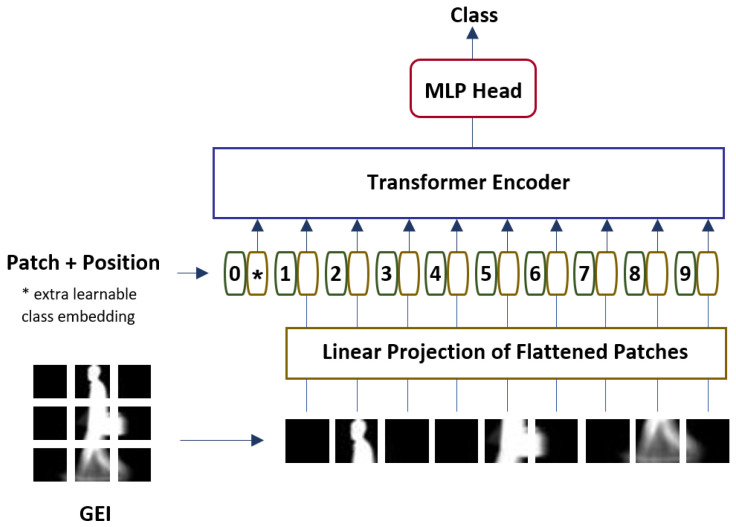
Architecture of the proposed Gait-ViT method.

This study presents a Vision Transformer model for gait recognition, known as Gait-ViT. Firstly, GEIs are acquired by averaging the gait images over a gait cycle. The attained GEIs are then converted into sequences of flattened 2D patches. Subsequently, the sequences of 2D patches are passed into the Vision Transformer model. In the Vision Transformer model, there are embedding layers, a Transformer encoder, and a multi-layer perceptron. The embedding layer converts the sequence of patches into an embedded patches vector by applying patch embedding. Thereafter, the positional embedding is added to the patch embedding to preserve the structure of the image patches. The embedded vectors are then fed into the Transformer encoder, where the final representation is determined. Lastly, the multi-layer perceptron performs classification based on the first token of the sequence. Figure 1 shows the architecture of the proposed Gait-ViT, which consists of an embedding layer, a Transformer encoder, and a multi-layer perceptron.

### 3.1. Gait Energy Image

**Figure 2:**
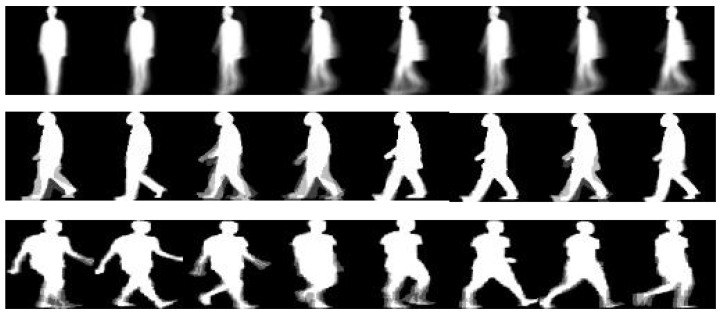
Instances of acquired GEIs, first row: CASIA-B, second row: OU-ISIR D, and last row: OU-LP.

Gait Energy Image (GEI) [41] conserves the shape and appearance of the human body and stride phase, which plays a significant role in the gait recognition procedure. The GEI is obtained by averaging the gait images over the gait cycle. The silhouette width of every frame in the gait video is collected into a time series signal. The local maximum in the time series signal corresponds to the frame when the two legs are furthest apart from each other. Alternatively, the width of the silhouettes reaches a local minimum when two legs wholly overlap. Each gait cycle is determined by alternate keyframes. In doing so, the effects of the noise and incomplete silhouettes are suppressed, which enhances the performance. Other than that, GEIs are grayscale images, which involve fewer parameters and require lower computational costs than the color images. As gait recognition is performed by focusing on the body movements, the information on the grayscale images is considered adequate. GEI is acquired using the weighted average method as below:

(1) $$x=\frac{1}{F}\sum_{f=1}^{F}I_{f}$$

where *F* is the total number of frames in a gait cycle and $I_{f}$ is the gait silhouette at frame *f*. Samples of GEIs are depicted in Figure 2.

### 3.2. Vision Transformer for Gait Recognition (Gait-ViT)

In the self-attention mechanism, every pixel attends to every other pixel. In other words, the pixels of a sequence interact with each other and identify which pixel they should pay more ‘attention’ to. The self-attention mechanism is mostly preferred because it disregards the vanishing gradient problem, as there is a direct connection between the encoder and decoder.

Due to the interaction among every pixel, the mechanism is costly to be applied to the images because of the huge number of pixels. This work leverages the Vision Transformer (ViT) model [42], where the image is split into a number of patches and converted into a sequence of image patches. The ViT model has several variants with different models and patch sizes. The ViT model was trained on ImageNet and Imagenet-21k datasets. The ViT adopted the Transformer model, which was presented by Vaswani et al. (2017) [43].

At first, the GEI is divided into fixed-size patches to reshape the GEI $x\in R^{H\times W\times C}$ into a sequence of flattened 2D patches $x_{p}\in R^{N\times \left(P^{2}\cdot C\right)}$ where *H* is the height, *W* is the width of the input image, *C* is the number of channels and (P,P) is image patch resolution. The number of patches *N* is calculated as below:

(2) $$N=\frac{H\times W}{P^{2}}$$

*N* also denotes the sequence length to be fed into the Transformer. By dividing the image into patches, the quadratic cost in the number of pixels is significantly reduced.

#### 3.2.1. Linear Projection of Flattened Patches

**Figure 3:**
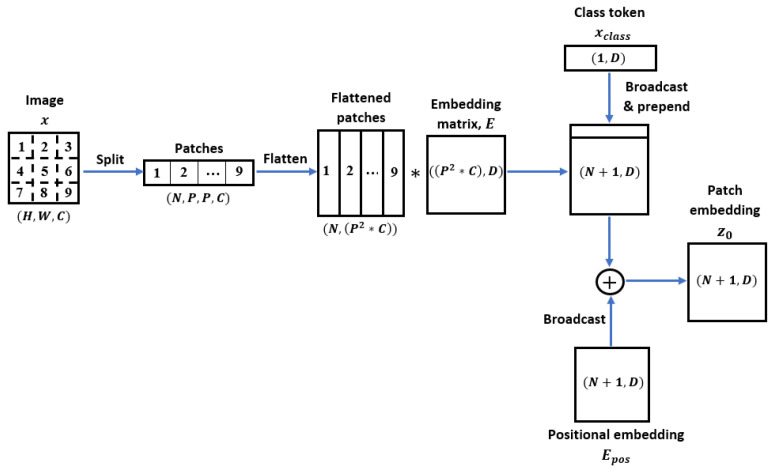
The process flow of patch embedding.

Before feeding the sequence of patches into the Transformer, the patches undergo a linear projection. During the linear projection, the patches are mapped into a vector of *D* dimension, where the vectors are multiplied by the embedding matrix E. The output of the linear projection is referred to as patch embeddings. To allow the model to encode the structure of the image, positional information $E_{pos}$ is appended to the patch embedding. Subsequently, the embedded image patches are concatenated along with a learnable class token $x_{\mathrm{class}}$, which is essential for the classification process. The initial patch embedding $z_{0}$ that consists of an embedded sequence of image patches with the class token is computed as stated below:

(3) $$z_{0}=\left[x_{\mathrm{class}\;};x_{p}^{1}E;x_{p}^{2}E;\cdots ;x_{p}^{N}E\right]+E_{pos},\;E\in R^{\left(P^{2}\cdot C\right)\times D},E_{pos}\in R^{(N+1)\times D}$$

where $x_{p}^{n}$ is the *n*-th image patch and n∈{1,2,…,N}. The obtained embedded image patches are then passed to the Transformer encoder. Figure 3 illustrates the generation process of the patch embeddings.

#### 3.2.2. Transformer Encoder

**Figure 4:**
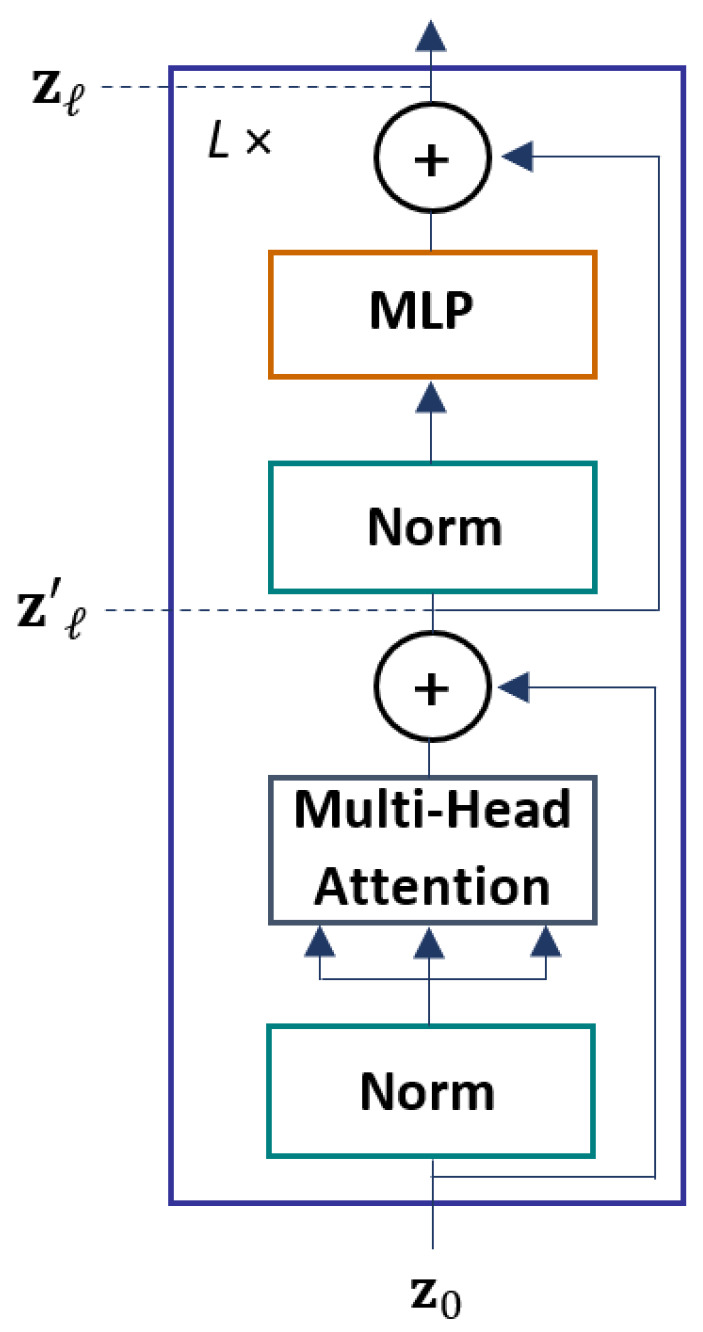
The architecture of the Transformer encoder.

The Transformer encoder comprises *L* identical encoder blocks. There are two sub-layers in every encoder block: a multi-head self-attention (MSA) and a fully connected feed-forward multi-layer perceptron (MLP), as depicted in Figure 4.

The *ℓ*-th encoder layer receives input sequence from the previous layer $z_{\ell -1}$. The input $z_{\ell -1}$ first goes through layer normalization. The layer normalization normalizes the input values for all the neurons across the feature dimension, which reduces the training time and enhances the performance. Subsequently, the output of the layer normalization is fed into the MSA layer.

The output of the MSA layer then goes through layer normalization again. Finally, the output from the layer normalization is sent to the MLP layer. In the encoder layer, the residual connections (also known as skip connections) are used to pass the information between disconnected layers. The residual connections allow the gradients to flow through the network without passing non-linear activation functions. By avoiding the non-linear activation functions, the vanishing gradient problem is prevented. The gradient flow in the *ℓ*-th encoder layer is defined as:

(4) $$z_{\ell }^{\prime }=\mathrm{MSA}\left(\mathrm{LN}\left(z_{\ell -1}\right)\right)+z_{\ell -1},\;\;\ell =1\ldots L$$

(5) $$z_{\ell }=\mathrm{MLP}\left(\mathrm{LN}\left(z_{\ell }^{\prime }\right)\right)+z_{\ell }^{\prime },\;\;\ell =1\ldots L$$

where LN denotes the layer normalization.

- Processes in Multi-head Self-Attention (MSA)

**Figure 5:**
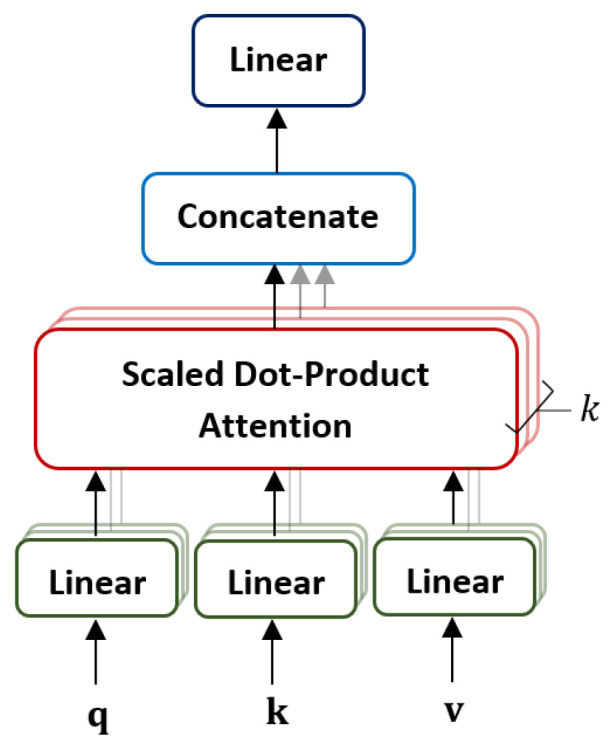
The architecture of the multi-head self-attention layer.

The MSA is made up of a linear layer, a self-attention layer, a concatenation layer, and a final linear layer, as depicted in Figure 5.

In MSA, multiple self-attention operations are conducted in parallel based on the number of heads *k*. In every head, the *D*-dimensional patch embedding z is multiplied with three weight matrices $U_{q}$, $U_{k}$, $U_{v}$ to produce three matrices: query (q), key (k), and value (v). The multiplication operation in every head is defined as below:

(6) $$\left[q,k,v\right]=\left[zU_{q},zU_{k},zU_{v}\right],\;\;U_{q},U_{k},U_{v}\in R^{D\times D_{h}}$$

The obtained matrices q,k,v are then mapped into *k* subspaces, where the weighted sum over all values *V* is calculated. The attention weights are then computed in every head based on the relationship among two elements (i,j), where the dot product is calculated based on $q^{i}$ and $k^{j}$. The output of the dot product shows the significance of the patches in the sequence. The dot product of the q and k is computed and the softmax function is applied to attain the weights on the values as below:

(7) $$A=\mathrm{softmax}\left(\frac{qk^{⊺}}{\sqrt{D_{h}}}\right),\;\;A\in R^{N\times N}$$

where $D_{h}=D/k$.

The difference between the standard dot-product operation and the self-attention (SA) dot-product operation is the usage of the dimension of the key $\frac{1}{\sqrt{D_{h}}}$ as a scaling factor, hence known as the scaled dot-product. Lastly, the output of the softmax is multiplied by the value v of each patch embedding vector to determine the patch with the highest attention score as below:

(8) SA(z)=Av

The self-attention matrices are then concatenated and mapped through a single linear layer with learnable weight $U_{msa}$ as shown:

(9) $$\mathrm{MSA}\left(z\right)=\left[{\mathrm{SA}}_{1}\left(z\right);{\mathrm{SA}}_{2}\left(z\right);\cdots ;{\mathrm{SA}}_{k}\left(z\right)\right]U_{msa},\;\;U_{msa}\in R^{k\cdot D_{h}\times D}$$

Every head of MSA captures information from different aspects at different positions, which allows the model to encode more intricate features in parallel. Furthermore, the computational cost of MSA is similar to a single head attention due to the parallel mechanism.

- Processes in Multi-layer Perceptron (MLP)

**Figure 6:**
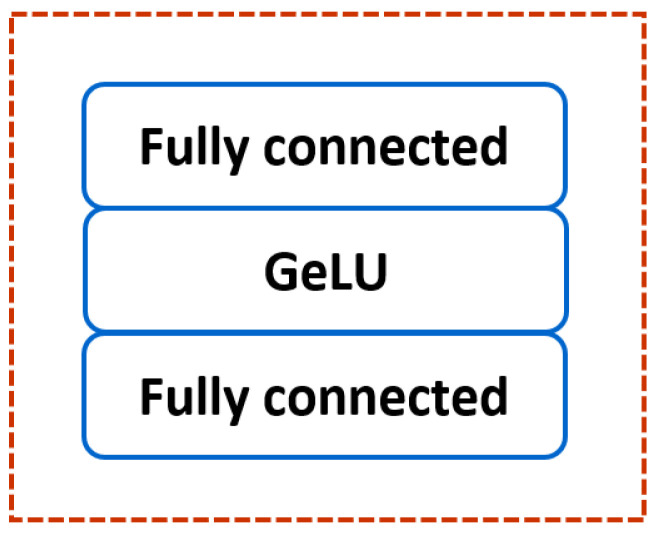
The architecture of the multi-layer perceptron.

The MLP comprises two fully connected layers with Gaussian Error Linear Unit (GeLU) activation function, as shown in Figure 6.

The GeLU function weighs inputs by their value rather than their sign. Contrary to the ReLU function, the GeLU function can be positive or negative, which has greater curvature. Therefore, the GeLU function can estimate complicated functions better than the ReLU function.

The last layer of the encoder picks the first token of the sequence $z_{L}^{0}$ and generates the image representation r by performing layer normalization. The r is then sent to a small MLP head which is a single hidden layer with a sigmoid function to perform the classification. The image representation of the sequence is obtained by:

(10) $$r=\mathrm{LN}\left(z_{L}^{0}\right)$$

During the training process, the Adam optimizer is applied to speed up the network convergence. The early stopping technique is used in the model to avoid over-training the network. The early stopping technique stops the network training process when there are no improvements in the validation set accuracy. Consequently, the categorical cross-entropy loss function is adopted in the proposed method due to the involvement of multi-class classification. The cross-entropy loss function is computed by:

(11) $$\mathrm{loss}=-\sum_{t=1}^{T}y_{t}\cdot \log{\hat{y}}_{t}$$

where ${\hat{y}}_{t}$ is the corresponding target value, $y_{t}$ is the prediction, and *T* is the number of test samples.

## 4. Experiments and Discussion

The datasets employed to assess the performance, hyperparameter settings, and performance of the proposed and existing methods are discussed in this section. The size of the obtained GEIs is set to H=64 and W=64. As the GEIs are grayscale images, the number of channels C=1. For the ViT model, the ‘Base’ variant (ViT-B/32) with patch size P=32 is adopted. The patch size 32 × 32 is chosen as patch size 16 × 16 requires a higher computational cost due to the longer sequence. With these settings, there are $N=\frac{64\times 64}{32^{2}}=4$ patches. The MSA consists of L=12 encoder layers and k=12 heads in each layer. As for the early stopping, the observation metric is the validation accuracy, and the patience is set to 15. Hence, when the validation accuracy of the model stops improving after 15 epochs, the model training is halted, and the best weights are restored. All the experiments are conducted on the Anaconda platform with NVIDIA GeForce RTX 2080 Ti.

### 4.1. Datasets

The performance evaluation of the proposed Gait-ViT is conducted on three gait datasets, namely the CASIA-B dataset, OU-ISIR dataset D, and OU-LP dataset.

The CASIA-B dataset [44] was built with 124 subjects where the gait sequences were recorded from 11 views. The sequences were captured based on the viewing angle, clothing, and carrying condition. The gait sequences were also recorded under normal walking, with coats and bags on.

The OU-ISIR dataset D [45] contains 185 subjects with 370 sequences. The gait sequences were captured with various gait fluctuation, which was grouped as DB${}_{low}$ and DB ${}_{high}$. Both sets comprise 100 subjects, each with stable walking (DB${}_{high}$) and fluctuated walking (DB${}_{low}$).

The OU-LP dataset [46] consists of 4016 subjects with an age range of 1 to 94 years old. The dataset contains sequence A and sequence B, where every subject has two sequences in sequence A, while one sequence is in sequence B. The gait sequences were recorded according to 55°, 65°, 75°, and 85° viewing angles. Sequence A with 3916 subjects is used in this work. A summary of the datasets is presented in Table 1.

### 4.2. Hyperparameter Tuning

There are four hyperparameters entail in the proposed method, namely, batch size *B*, learning rate *R*, input size *I*, and optimizer θ. A grid search technique is applied manually on the CASIA-B dataset to tune the aforementioned hyperparameters. To perform the grid search, the value of a specific hyperparameter is modified, while the values of the remaining hyperparameters are kept unchanged.

The accuracy of the proposed method at various batch sizes *B* are shown in Table 2. The highest accuracy is acquired at batch size 32. The larger batch sizes not only require high computational costs but also lead to poor generalization. Other than that, the larger batch sizes induce lower asymptotic accuracy.

Table 3 displays the accuracy of the proposed method at different learning rates *R*. The learning rate at 0.0001 achieved the highest accuracy. The lower learning rate causes overfitting issues and also slows down the training, thus wasting the computational resources with no progress in the accuracy. On the contrary, the larger learning rate converges too fast, thus, disregarding the optimal solution.

The accuracy of the proposed methods at various input sizes *I* are illustrated in Table 4. The highest accuracy is obtained at input size 64 × 64. Although the smaller input size involves low computational cost, it might lose some of the crucial information. On the other hand, the larger input size could have more irrelevant information and requires high computational cost.

Table 5 presents the accuracy of the proposed method using different optimizers θ. As the CASIA-B dataset contains incomplete and noisy silhouettes, Adam optimizer performs promisingly compared to SGD and Nadam. This is due to the capability of the Adam optimizer to handle noisy and sparse gradients. Furthermore, both the SGD and Nadam utilize higher computational time than the Adam optimizer.

The optimal values for the hyperparameters are chosen considering the accuracy and time consumption. Table 6 displays the tested values and optimal values of hyperparameters for the proposed Gait-ViT method.

### 4.3. Comparison with the Existing Methods

To compare the performance of the proposed Gait-ViT method, five state-of-the-art methods are included in this work, namely GEINet [47], Deep CNN [48], CNN with Leaky ReLU [49], CNN [50], and deep CNN [51]. The datasets are segregated into three sets as 80% training, 10% validation, and 10% testing. The input size is set to 64 × 64 for all the existing methods to have an unbiased assessment. The accuracy of the proposed Gait-ViT method and the existing methods on different datasets are shown in Table 7.

The accuracy of the majority of the existing methods, particularly the deep CNN method [48] decreased in the CASIA-B dataset due to the incomplete and noisy silhouettes. However, the proposed Gait-ViT method surpasses the existing methods by obtaining an accuracy of 99.93%. This is due to the multi-head attention mechanism that focuses and incorporates the information through the whole image, where each of the attention heads attends to different regions of the image. The concatenation of the various information from every attention head, along with the dynamic, receptive field and strong residual connection, improves the ability to handle incomplete and noisy silhouettes.

Using the OU-ISIR dataset D, all the methods yield higher accuracy as the dataset comprises a small number of subjects. The proposed Gait-ViT method obtained 100% in both DB${}_{high}$ and DB${}_{low}$. The Transformer has the ability to adjust the receptive field based on the nuisances in the input image, which results in a flexible and dynamic receptive field. This characteristic makes the Transformer robust to occlusion and slight variation in shapes. Hence, the proposed method achieved high accuracy, regardless of the slight variation in the walking shapes.

As for the OU-LP dataset, the accuracy of the [48,50,51] methods are quite low due to a large number of subjects. Nonetheless, the proposed method performed promisingly with an accuracy of 99.51%, which shows the improvement in the generalization of the proposed method. The Transformer is a global operation where the global interactions among other patch embeddings take place. By doing so, local information of the image is encoded, as well as modeling the global relationship between distant image parts. Furthermore, the global operation contributes to conserving the structural information of the patch sequence. Hence, the incorporation of the Transformer enhances the generalization ability of the proposed Gait-ViT method.

## 5. Conclusions

This paper presents a work with a vision transformer for gait recognition. The gait energy image is first computed by averaging the series of gait silhouettes over a gait cycle. The obtained gait energy image is then split into fixed-size patches. Subsequently, patch embedding and positional embedding are applied to the patches during the linear projection, where the patches are flattened into a long vector with information about the position of every vector. The flattened and embedded vector is then fed to the Transformer to generate the final gait representation. Lastly, the classification is performed by transforming the first token of the sequence into a class prediction. The experimental results exhibit the scalability of the ViT in handling both small and large datasets. Moreover, the proposed method shows robustness toward noisy and incomplete silhouettes with the application of the self-attention mechanism. The multi-head attention mechanism, which works in parallel, contributes to the improvement of the parallelization ability. Other than that, the residual connection, layer normalization, and early stopping enhance the performance as well as reduce the computational cost. As the datasets used in the experiment consist of various covariates, which resemble real-world scenarios, the ViT model is adept at identifying the subject regardless of the covariates. The limitation of the ViT model is that the model consists of only one convolutional layer, where it cannot extract features from a larger input size, which contains more information than the smaller input sizes. Future work will be focused on enhancing the ViT architecture by adding more convolutional layers for better feature extraction.

## Figures and Tables

**Table 1:** Summary of datasets.

Datasets	Number of Subjects	Sequences	Angle Views	Variations
CASIA-B	124	10	11	Normal walking, Carrying condition, Clothing
OU-ISIR DB${}_{low}$	100	370	1	Steady walking
OU-ISIR DB${}_{high}$	100	370	1	Fluctuated walking
OU-LP (Sequence A)	3916	2	4	4 viewing angles

**Table 2:** Accuracy at different batch sizes *B* [*I* = 64 × 64, *R* = 0.0001, θ = Adam].

Batch Size	Accuracy (%)	Training Time (s)
32	99.93	2555.7536
64	99.41	739.3975
128	99.34	621.2719

**Table 3:** Accuracy at different learning rates *R* [*B* = 32, *I* = 64 × 64, θ = Adam].

Learning Rate	Accuracy (%)	Training Time (s)
0.00001	99.34	2259.2996
0.0001	99.93	2555.7536
0.001	99.41	3025.1851
0.01	43.86	1881.2865

**Table 4:** Accuracy at different input sizes *I* [*B* = 32, *R* = 0.0001, θ = Adam].

Input Size	Accuracy (%)	Training Time (s)
32 × 32	99.34	600.9491
64 × 64	99.93	2555.7536
128 × 128	98.60	2838.1245

**Table 5:** Accuracy at different optimizers θ [*B* = 32, *I* = 64 × 64, *R* = 0.0001].

Optimizer	Accuracy (%)	Training Time (s)
SGD	76.89	9449.4783
Adam	99.93	2555.7536
Nadam	99.63	3781.5499

**Table 6:** Summary of optimal hyperparameters for the proposed Gait-ViT method.

Hyperparameters	Tested Values	Optimal Value
Batch Size	32, 64, 128	32
Learning Rate	0.00001, 0.0001, 0.001, 0.01	0.0001
Input Size	32 × 32, 64 × 64, 128 × 128	64 × 64
Optimizer	SGD, Adam, Nadam	Adam

**Table 7:** Comparison results on different datasets.

Methods	Accuracy (%)			
	CASIA-B	OU-ISIR DB${}_{\mathrm{high}}$	OU-ISIR DB${}_{\mathrm{low}}$	OU-LP
GEINet [47]	97.65	99.93	99.65	90.74
Deep CNN [48]	25.68	87.70	83.81	5.60
CNN [49]	98.09	99.65	99.37	89.17
CNN [50]	94.63	89.99	96.73	48.32
Deep CNN [51]	86.17	96.18	95.21	45.52
Gait-ViT	99.93	100.00	100.00	99.51

## Data Availability

Not applicable.
